# Central retinal artery occlusion in a young child affected by COVID-19: a first case report

**DOI:** 10.1186/s12887-023-04276-8

**Published:** 2023-09-13

**Authors:** Giulia Abbati, Camilla Fazi, Pina Fortunato, Sandra Trapani

**Affiliations:** 1 Pediatric and Neonatology Unit, Santo Stefano Hospital, Prato, Italy; 2 Pediatric Unit, Meyer Children’s Hospital IRCCS, Florence, Italy; 3 Department of Health Sciences, University of Florence, Florence, Italy; 4 Pediatric Ophthalmology Unit, Meyer Children’s Hospital IRCCS, Florence, Italy

**Keywords:** Central retinal artery occlusion (CRAO), Optic nerve, SARS-CoV-2, Pediatric, Case report

## Abstract

**Background:**

Central retinal artery occlusion (CRAO) is an ophthalmic emergency, and its etiology is generally ascribed to vessel occlusion by a thrombus or embolus, eventually due to a hypercoagulable state. CRAO occurrence is described even in the pediatric population, but its incidence is very rare. SARS-CoV-2 infection has a multitude of presentations, and almost any organ may be involved including the ocular district. Cases of CRAO in patients affected by COVID-19 are reported in the literature in the adult population, but not in the pediatric one.

**Case presentation:**

We describe the case of a six-year-old otherwise healthy girl, who presented a sudden and complete bilateral vision loss after a one-day fever. All the clinical, ophthalmological, laboratory and instrumental investigations led to the diagnosis of a right CRAO and the suspicion of a contralateral posterior optic nerve affection. These manifestations could not be ascribed to any etiological condition apart from the documented ongoing mild SARS-CoV-2 infection. Treatment with anticoagulants and steroids was tried but the visual outcome was poor during the one-month hospitalization and at the last follow-up.

**Conclusions:**

To the best of our knowledge, this is the first report of CRAO in the course of SARS-CoV-2 infection in the pediatric age. In our review of the literature, we found few cases of CRAO in adults with COVID-19; we highlighted differences in anamnestic, clinical, and interventional aspects and therefore we tried to summarize the state of the art on this topic to facilitate further studies. Even if rare, the prognosis of CRAO is poor and the thrombolytic treatment could be effective only if rapidly administered, so the disease suspicion should be high in a patient with sudden vision loss, also in pediatric age.

## Background

Sudden blindness is a rare but dramatic condition both in adults and children. Optic nerve abnormalities are the most important cause of blindness in children; however, several acquired retinal diseases should be considered too. Central retinal artery occlusion (CRAO) is a rare event in the pediatric population, but it may be responsible for vision loss. Its etiology is generally ascribed to hypercoagulable states or embolic events, even if scientific literature reports some other less common causes of CRAO as well [1]. Since the first few months of the severe acute respiratory syndrome type 2 Coronavirus (SARS-CoV-2) pandemic, it became clear that this virus may not only provoke respiratory symptoms but even a potential multi-organ involvement. It is also widely known that the pro-inflammatory condition caused by SARS-CoV-2 is associated with a pro-coagulant state which predisposes the patients to thromboembolic events [2]. The ocular district is not spared by these manifestations and few cases of retinal or ophthalmic vessels occlusion are reported in adults affected by COVID-19 [3–13]. Nevertheless, to the best of our knowledge, no case has been previously reported in the pediatric age. In this paper, we describe the first case of CRAO in a young child who suffered bilateral vision loss during SARS-CoV-2 infection. This patient was already included in a recently published pediatric series [14], but we think this unique case is worthy of further details.

## Case presentation

A previously healthy 6-year-old Moroccan girl, feverish for a day, presented to the Emergency Department (ED) of a secondary-level hospital complaining of a sudden, painless, and complete bilateral vision loss, noticed about one hour earlier, upon awakening. Family and past personal medical histories were negative, no history of trauma or other acute events was reported, except for the recent fever, and the girl was not vaccinated for SARS-CoV-2. Once admitted, the nasal swab resulted positive for SARS-CoV-2 antigen. A first ophthalmologic examination showed bilateral nonreactive mydriasis and a visual acuity limited to *hand motion* at 30 cm in the left eye and *no light perception* in the right eye. At the fundus oculi exam, the left eye appeared ophthalmoscopically normal whereas pathognomonic findings of CRAO (white ischemic retina and a cherry-red spot in the foveal region) were revealed in the right eye. The remaining clinical examination, including a detailed neurologic evaluation, was normal. Radiological imaging of the brain (i.e., CT, MRI and MRA) did not reveal any abnormalities.

**Fig. 1:**
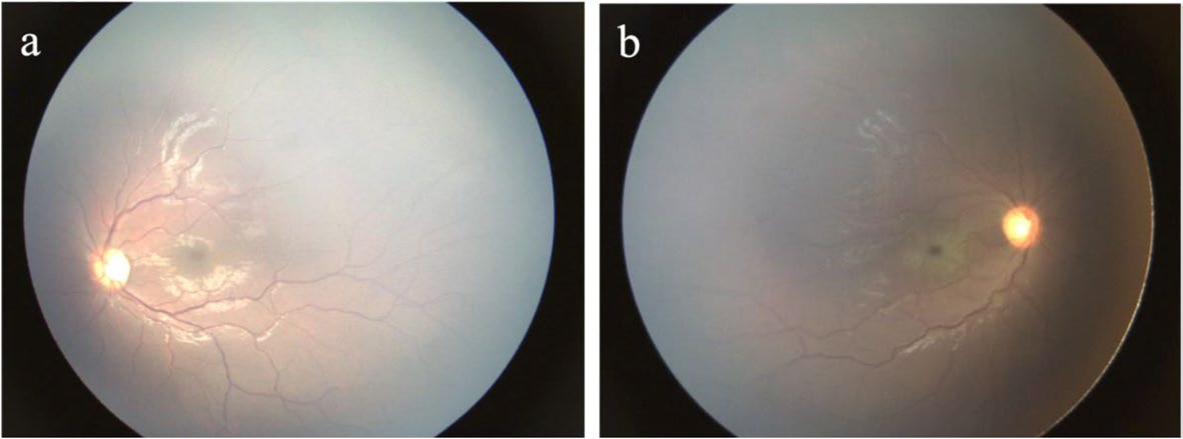
Fundus photographs at hospital admission. In the left eye the macula and optic nerve appeared normal (**a**); in the right eye the examination showed a cherry-red spot at the macula with a background of retinal whitening (**b**)

After her immediate transfer to the tertiary-level Meyer Children’s University Hospital, the ophthalmologic assessment confirmed the right CRAO (Fig. 1) and suspected a left posterior optic nerve affection.

The optical coherence tomography (OCT) showed a bilateral macular thickness reduction, even more in the right eye (183 μm) than in the left one (185 μm).

**Fig. 2:**
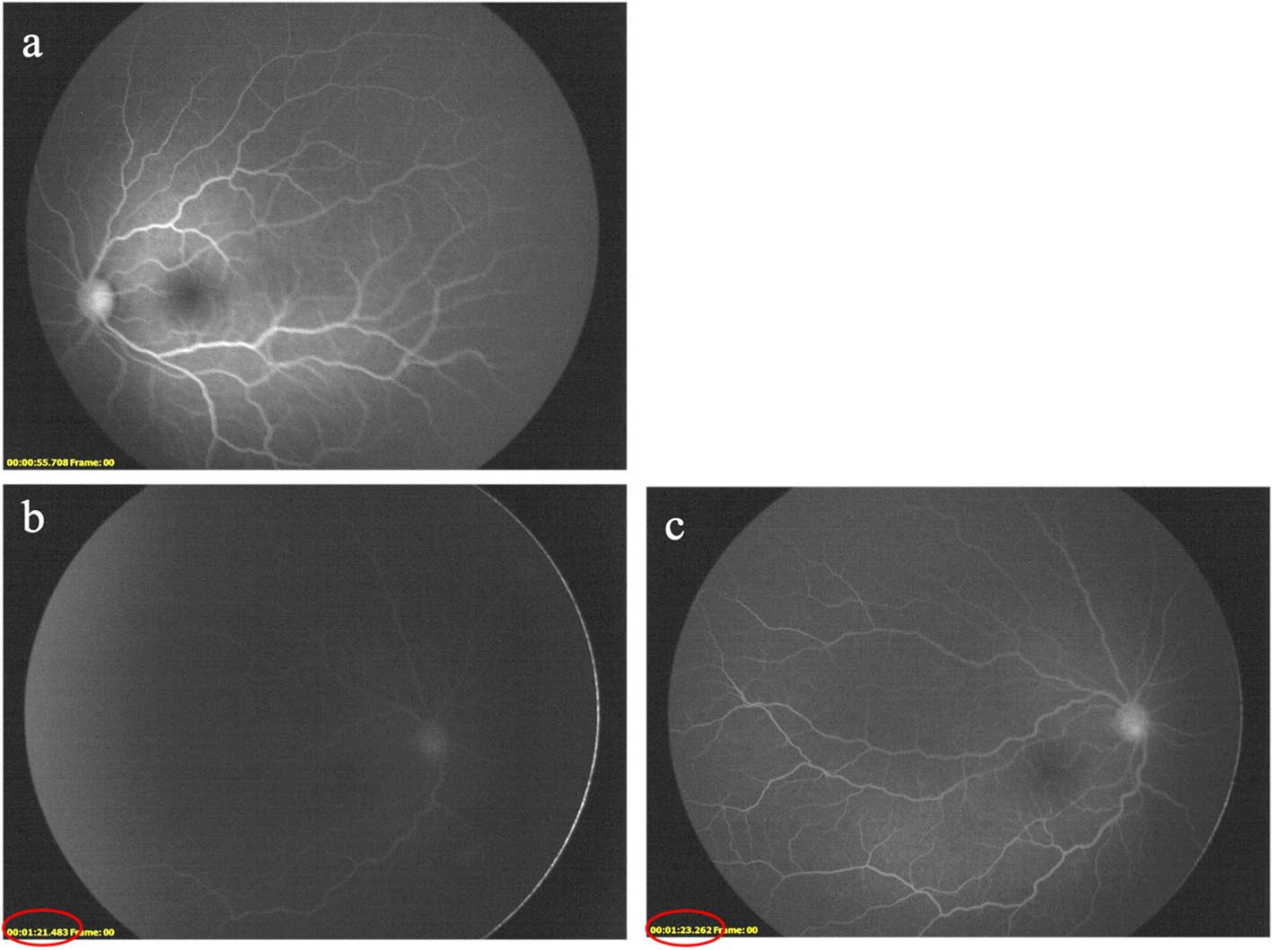
Fluorescein angiography (FA) 6 days after the onset of visual loss. In the left eye no significant alterations were appreciated 55 s after injection of fluorescein dye (**a**); in the right eye a delayed retinal arteriovenous transit time of sodium fluorescein was evident (*red circles*), together with attenuated retinal arteries, representing possible features of vascular damage (**b** and **c**)

During the following days, the fluorescein angiography (FA) confirmed the right CRAO, revealing poor and delayed blood flow in the right eye circulation (Fig. 2). Flash visual evoked potentials (VEP) supported the hypothesis of a left optic neuropathy showing a reduced left optic nerve conduction velocity (P100 latency 150 ms), with a preserved right one (P100 latency 101 ms).

Considering that symptoms started more than six hours earlier, thrombolytic treatment was excluded. Anticoagulant therapy (100 mg/kg/day low molecular weight heparin [LMWH]) was therefore initiated, together with intravenous (iv) steroids (30 mg/kg/day methylprednisolone) for three days followed by oral prednisone (2 mg/kg/day).

Meanwhile, a new brain MRI (T2-weighted, FLAIR and 3D T1-weighted contrast-enhanced, DWI and SWI sequences), together with orbits MRI (axial T2-weighted and FLAIR, coronal STIR, 3D T1-weighted contrast-enhanced and Dixon sequences), resulted normal, also excluding any morphological alteration or signal change in the optic nerves and gadolinium-enhancing. Even a digital subtraction angiography (DSA) was performed, and no vessel alterations have been documented.

All the performed blood exams, including inflammatory and coagulation markers (Table 1), hemoglobin electrophoresis, extensive thrombophilic and autoimmune screening, metabolic and infectious tests, resulted negative, except for the positivity of antinuclear antibodies (ANA) with a 1:640 titer and SARS-CoV-2 serology (IgG against SARS-CoV-2 spike glycoprotein S1 and nucleocapsid protein). The presence of SARS-CoV-2 was confirmed on the nasal swab even by the polymerase chain reaction (PCR) technique.

**Table 1:** Main laboratory findings at hospital admission

**Marker**	**Value**	**Normal range**
CRP [mg/dl]	1.12	0.00 – 0.50
ESR [mm/h]	26	2 – 31
Ferritin [ng/ml]	144	15–300
WBC count [cells/μl]	8.000	3.500 – 14.000
Neutrophils [%]	48.3	35 – 70
Lymphocytes [%]	43.4	20 – 60
PT [%]	100	70 – 100
aPTT [s]	24	30 – 35
I.N.R. [U]	1.00	0.87 – 1–20
Fibrinogen [mg/dl]	370	200 – 410

*aPTT* Activated partial thromboplastin time, *CRP* C-reactive protein, *ESR* Erythrocyte sedimentation rate, *I.N.R.* International normalized ratio, *PT* Prothrombin time, *WBC* White blood cell

In addition, an eventual cardiac affection was excluded by performing an electrocardiogram and echocardiography.

Three weeks later, LMWH was switched to acetylsalicylic acid (ASA) provided at the anti-thrombotic dose, and steroid therapy was gradually tapered. These therapies were interrupted after four and five months, respectively.

During the one-month hospitalization period, regular ophthalmologic assessments were performed but visual impairment resulted substantially unmodified despite the treatment and optic disc pallor developed in both eyes. Therefore, at discharge, the patient was pointed toward a learning path for visually impaired people.

**Fig. 3:**
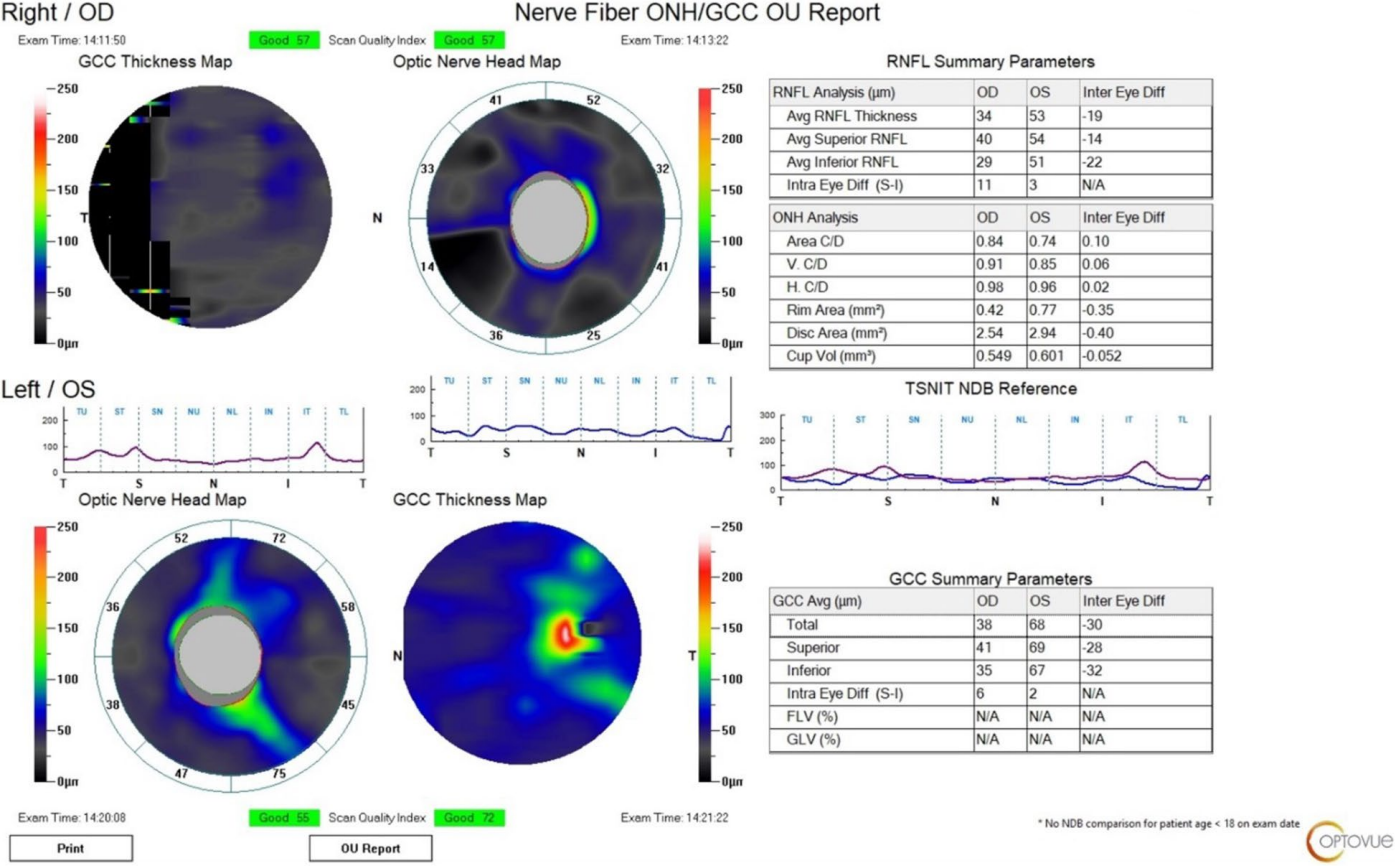
The optical coherence tomography (OCT) performed at the 5-month (last) follow-up showed a reduced thickness of the retinal nerve fiber layer (RNFL) in both eyes

Throughout the ophthalmological follow-up, the girl showed a mild left-eye visual recovery with light perception and the capability of counting fingers at 30 cm. Nevertheless, right-eye blindness persisted without any improvement, as well as the extreme bilateral reduction of the retinal nerve fiber layer (RNFL) at the OCT examinations at 3-month and 5-month follow-ups (Fig. 3).

## Discussion and conclusions

CRAO is an ophthalmic and medical emergency that provokes retinal hypoperfusion and that may be responsible for retinal infarction, which is considered an acute ischemic stroke (AIS) [15]. Patients typically present with profound, acute, and painless monocular visual loss, and they often develop a severe visual impairment [15, 16]. CRAO occurs primarily in the elderly, whereas it is an extremely rare diagnosis in children and young adults, with an estimated incidence lower than 1:50.000 people under 30 years old [17]. CRAO etiologies and risk factors are multifactorial and they include hypercoagulable states (hyperhomocysteinemia, hyperlipidemia, polycythemia, thrombocytosis, sickle cell disease, coagulation abnormalities, nephrotic syndrome, etc.), trauma, vasospasm, and vasculitis such as Giant Cells Arteritis in the elderly or those detected in systemic lupus erythematosus, tuberculosis, syphilis etc. [1, 16–19].

The relationship between CRAO and COVID-19 is still debated and CRAO could be a coincidence in infected patients; a review including 17 case reports in adulthood stated that the strength of data is insufficient to define the true correlation between retinal vascular occlusions and COVID-19 [20]. In our case, the link between SARS-CoV-2 infection and the occurrence of CRAO could be hypothesized considering that other possible underlying conditions were excluded by an accurate medical history, a complete physical examination, and an extensive workup. Indeed, apart from the documented ongoing SARS-CoV-2 infection, isolated ANA positivity was the only, although unspecific, finding. Besides, recent scientific literature suggests a possible association between SARS-CoV-2 vaccination and CRAO, along with other ocular vascular events [21, 22]. However, we could easily exclude this potential risk factor since our patient was not vaccinated. In the last few years some case reports about CRAO of unknown origin have been published and the authors concluded for idiopathic forms of CRAO [23, 24]; nevertheless, in our patient the temporal link between SARS-CoV-2 infection and CRAO is remarkable.

It is well known that SARS-CoV-2 infection has a multitude of presentations including ophthalmic manifestations, as accurately described by several authors [25, 26]. Although the pathogenetic mechanism has not been well established yet, the virus seems to induce a remarkable inflammatory response, with consequent endothelial dysfunction and an overall prothrombotic and hypercoagulable state which predisposes to thrombotic disease in both venous and arterial circulation [2, 3, 11, 26–28].

We conducted a detailed review of the literature, and as far as we know, no other cases of CRAO associated with SARS-CoV-2 infection have been documented in pediatric age. However, some cases have been described in adults, in addition to other ocular vascular occlusions [3–13, 25]. Kulkarni et al. presented a case of combined central retinal artery and vein occlusion (CRVO) in a 20-year-old boy [9]. For what concerns pediatric population, retinal vasculitis has been described in a child five months after an asymptomatic SARS-CoV-2 infection [29].

In most cases, the pathogenetic mechanism is attributed to the already cited hyperinflammatory and hypercoagulability state [3, 11], whereas Turedi et al. reported a case of CRAO without demonstrated laboratory alterations, including the hypercoagulability workup [8].

The girl we described developed the ocular manifestation very early in the COVID-19 course, in contrast with many other reports, where patients presented CRAO later during the hospitalization or even after the discharge [3, 5, 8, 9]. Moreover, unlike our report, adult patients with CRAO often presented a severe form of COVID-19, requiring hospitalization and sometimes intensive care unit (ICU) admission [3, 5, 6, 9, 13]; in addition, many of these patients had various risk factors for CRAO, being hypertension the most frequently reported [3–5, 7, 11, 13].

The exact time length of reversible retinal hypoxic damage is unknown yet and it is estimated between 4.5 and 12 h [15, 30]. Intravenous tissue plasminogen activator (tPA) is an evidence-based therapy for AIS, when provided within 4.5 h [15, 31, 32]. Treatment with tPA showed a strong effect even in patients with CRAO [33], however adequate randomized clinical trials are still ongoing [15]. In centers where endovascular therapy is performed, intra-arterial tPA (Ia-tPA) may be considered an alternative treatment, and the time window for drug administration is potentially increased [15]. Regretfully, although prompt management of acute CRAO is mandatory to maximize visual outcome and avoid any secondary ischemic event, a unanimous consensus on the topic has not been reached yet. Our child did not receive a thrombolytic treatment due to the late diagnosis, whereas anticoagulant therapy was soon administered. Several non-invasive treatments have been proposed and tried in some adult cases [4, 7, 8, 11, 13]; however these strategies are not uniformly supported by the current scientific literature, since there is no evidence of effectiveness [15, 16, 30]. In order to improve retinal oedema, and bearing in mind the known inflammatory and vasculitis tendency of SARS-CoV-2 infection, iv and oral steroids were administered to our patient, nonetheless with poor visual outcome, in accordance to other adult cases in the literature [9, 12] and to the known prognosis of CRAO [3, 8].

Furthermore, it can be postulated that besides the right demonstrated CRAO, the viral infection was involved even in the contralateral eye affection. Considering the alteration of visual acuity and VEP in the left eye, with normal fundus examination, a damage of the posterior segment of the left optic nerve was supposed. A wide range of conditions may cause optic neuropathies, such as autoimmune diseases, trauma, vascular injuries, and infections [34]. It is well known that SARS-CoV-2 has neurotropic characteristics and numerous studies provided strong evidences for its neurovirulence [25, 35], as already known for other beta coronaviruses [35]. Several cases of optic neuropathies have been reported after COVID-19 and these complications may be severe [36]. Optic neuritis (ON) in confirmed SARS-CoV-2 infection has been described both in adult and pediatric population [25, 37, 38]. Otherwise ON was considered unlikely in our patient due to the clinical presentation (painless vision loss, normal fundus) and especially to the absence of optic nerves alterations at the orbit MRI (including T2-weighted and STIR sequences) [36]. Another considered hypothesis was the occurrence of a bilateral CRAO, probably a left transient CRAO with cilioretinal artery sparing but with damage of RNFL and optic nerve, as documented by the OCT performed five months after the insult (Fig. 3). In fact, structural injuries (in terms of thickness reduction in the macular and peripapillary RNFL) are evident in patients with retinal artery occlusion and a close correlation between RNFL thinning and functional outcome has been found [39]. Besides, significant changes in peripapillary and macular RNFL thickness have been described in children with recent COVID-19 [40].

In conclusion, our case is worthy of attention because it is the first pediatric case of CRAO temporarily linked to SARS-CoV-2 infection. CRAO is a rare entity in children but, as confirmed by our case, the prognosis may be poor. Therefore, pediatricians and ophthalmologists should always consider this condition in a child with sudden vision loss and investigate the possibility of a recent SARS-CoV-2 infection. Moreover, CRAO may be an early manifestation of COVID-19, and its usual predisposing factors may be lacking. Thrombolytic treatment seems to be effective when rapidly administered therefore the disease suspicion should be higher to arrive at prompt diagnosis and intervention. Finally, different pathogenetic hypotheses were made to explain the contralateral eye affection, being optic neuropathy the most probable. Therefore, we believe that further studies on the association between SARS-CoV-2 and visual impairments are needed because they may have a significant impact on children vision and quality of life.

## Data Availability

Not applicable.

## Abbreviations

AIS: Acute ischemic stroke
ANA: Antinuclear antibodies
aPTT: Activated partial thromboplastin time
ASA: Acetyl salicylic acid
COVID: Coronavirus Disease
CRAO: Central retinal artery occlusion
CRP: C-reactive protein
CRVO: Central retinal vein occlusion
CT: Computed tomography
DSA: Digital subtraction angiography
ED: Emergency department
ESR: Erythrocyte sedimentation rate
FA: Fluorescein angiography
FLAIR: Fluid attenuated inversion recovery
Ia-tPA: Intra-arterial tissue plasminogen activator
ICU: Intensive care unit
INR: International normalized ratio
iv: Intravenous
LMWH: Low-molecular weight heparin
MRA: Magnetic resonance angiography
MRI: Magnetic resonance imaging
OCT: Optical coherence tomography
ON: Optic neuritis
PT: Prothrombin time
RNFL: Retinal nerve fiber layer
SARS-CoV-2: Severe acute respiratory syndrome type 2 Coronavirus
STIR: Short tau inversion-recovery
tPA: Tissue plasminogen activator
VEP: Visual evoked potentials
WBC: White blood cell
